# Sponge community of the western Black Sea shallow water caves: diversity and spatial distribution

**DOI:** 10.7717/peerj.4596

**Published:** 2018-05-08

**Authors:** Alexander Ereskovsky, Oleg A. Kovtun, Konstantin K. Pronin, Apostol Apostolov, Dirk Erpenbeck, Viatcheslav Ivanenko

**Affiliations:** 1 Institut Méditerranéen de Biodiversité et d’Ecologie Marine et Continentale (IMBE), Aix Marseille University, CNRS, IRD, Avignon Université, Marseille, France; 2 Department of Embryology, Faculty of Biology, Saint-Petersburg State University, Saint-Petersburg, Russia; 3 Hydrobiology and General Ecology Department, Marine Research Station, Odessa National I. I. Mechnikov University, Odessa, Ukraine; 4 Physical and Marine Geology Department, Odessa National I. I. Mechnikov University, Odessa, Ukraine; 5 Burgas University, Burgas, Bulgaria; 6 Department of Earth and Environmental Sciences & GeoBio-Center, Ludwig-Maximilians-Universität München, Munich, Germany; 7 Department of Invertebrate Zoology, Biological Faculty, Moscow State University, Moscow, Russia

**Keywords:** Bulgaria, The Black Sea, Porifera, Marine caves, Checklist, Karst cave habitat, Extreme environment, Inventory, Cave-dwelling fauna

## Abstract

Marine caves possess unique biocoenotic and ecological characteristics. Sessile benthic species such as sponges associated with cave habitats typically show a marked zonation from the cave entrance towards the end of the cave. We describe three semi-submerged karstic caves of 50 to 83 m length and 936 to 2,291 m^3^ volume from the poorly explored cavernicolous fauna of North-East Bulgaria. We surveyed sponge diversity and spatial variability. Eight demosponge species were identified based on morphological and molecular data, of which six are known from the adjacent open sea waters of the Black Sea. Two species, *Protosuberites denhartogi* van Soest & de Kluijver, 2003 and * Halichondria bowerbanki* Burton, 1930, are reported from the Black Sea for the first time. The spatial sponge distribution inside the caves is in general similar, but shows some differences in species composition and distribution depending on cave relief and hydrodynamics. The species composition of sponges of Bulgarian caves is found to be different from Crimean caves. An updated checklist of the Black Sea sponges is provided.

## Introduction

During the last decades it has repeatedly been shown that the environmental conditions in submarine caves produce a rich and diversified biota, and bear several faunistic peculiarities including ‘relict’ species (Hart, Manning & Iliffe, 1986; Ohtsuka, Hanamura & Kase, 2002; Manconi, Serusi & Pisera, 2006; Manconi et al., 2009; Manconi & Serusi, 2008; Iliffe & Kornicker, 2009; Pisera & Vacelet, 2010), bathyal forms (Harmelin, 1997; Vacelet, 1996; Glover et al., 2010; Janssen, Chevaldonné & Martínez Arbizu, 2013), and truly troglobial taxa (Pansini, 1996; Bussotti et al., 2006; Chevaldonné & Lejeusne, 2003; Lejeusne & Chevaldonné, 2005). These faunas are composed of species able to tolerate the cave conditions and possess adaptive colonization strategies. Moreover, for meiofauna (Todaro et al., 2006; Janssen, Chevaldonné & Martínez Arbizu, 2013) as well as for macrofauna, the marine caves represent hotspots of biodiversity and endemicity (Gerovasileiou & Voultsiadou, 2012). Submerged or partially submerged sea caves are protected by the EU Habitats Directive as a distinct habitat type (UNEP-MAP-RAC/SPA, 2008; Giakoumi et al., 2013).

In contrast to submerged marine caves, semi-submerged caves are characterized by higher hydrodynamics, possess insenvironmental conditions on short and long term, and are rarely oligotrophic (Corriero et al., 2000). Light and water-movement variations are related to the peculiar cave characteristics in terms of morphology, dimensions, exposure, and depth. In the shallow semi-submerged, the rapid decrement of light intensity may not correspond to a similar decreasing trend of water-movement. It has been shown that more illuminated sections in caves and shallow and/or semi-submerged caves are more susceptible to marine biological invasions than darker sections (Gerovasileiou et al., 2016; Dimarchopoulou, Gerovasileiou & Voultsiadou, 2018).

Sponges (Phylum Porifera) represent one of the most abundant animal phyla in many benthic ecosystems (Bell & Smith, 2004) and can constitute the dominant sessile organism group in marine cave environments (Sarà, 1962; Corriero et al., 2000; Richter et al., 2001; Bell, 2002; Gerovasileiou & Voultsiadou, 2012; Gerovasileiou & Voultsiadou, 2016; Gerovasileiou et al., 2016; Radolović et al., 2015; Ereskovsky, Kovtun & Pronin, 2016). The sponge fauna of marine semi-submerged caves has been investigated predominantly in the Mediterranean Sea (Sarà, 1958; Sarà, 1961a; Sarà, 1961b; Laborel & Vacelet, 1959; Labate, 1964; Vacelet, 1976; Vacelet, 1996; Pansini & Pronzato, 1982; Corriero et al., 1997; Corriero et al., 2000; Corriero, 1989; Knittweis et al., 2015; Dimarchopoulou, Gerovasileiou & Voultsiadou, 2018), although a few studies were performed in Ireland (Bell, 2002), in the Caribbean area (Macintyre et al., 1982; Hart, Manning & Iliffe, 1986; Kobluk & Van Soest, 1989), and the Black Sea (Ereskovsky, Kovtun & Pronin, 2016). Here, it was clearly demonstrated that superficial caves do not resemble submerged caves in terms of benthos diversity and community structure. As submarine caves (submerged and semi-submerged) are subject to severe temperature alterations attributed to global climate change (Bianchi, Morri & Russo, 2003), as well as to biological invasions (Gerovasileiou et al., 2016) and anthropogenic impacts (Nepote et al., 2017), knowledge on the Black Sea sponge diversity is vital. Submarine caves represent poorly resilient ecosystems (Harmelin, 1980; Lejeusne & Chevaldonné, 2006), therefore, understanding their recovery capacity after disturbances is mandatory for conservation.

Our knowledge on the biodiversity of the Black Sea sponges is scarce; data describing the sponge diversity in its caves is practically non-existent, except for one publication on Crimean cave sponges (Ereskovsky, Kovtun & Pronin, 2016).

The objectives of the present study are the description of three shallow water semi-submerged marine caves explored in the Bulgarian Black Sea coast, including the assessment of their sponge diversity and distribution as groundwork for the environmental management of coastal zones in the Black Sea.

## Material & Methods

**Figure 1:**
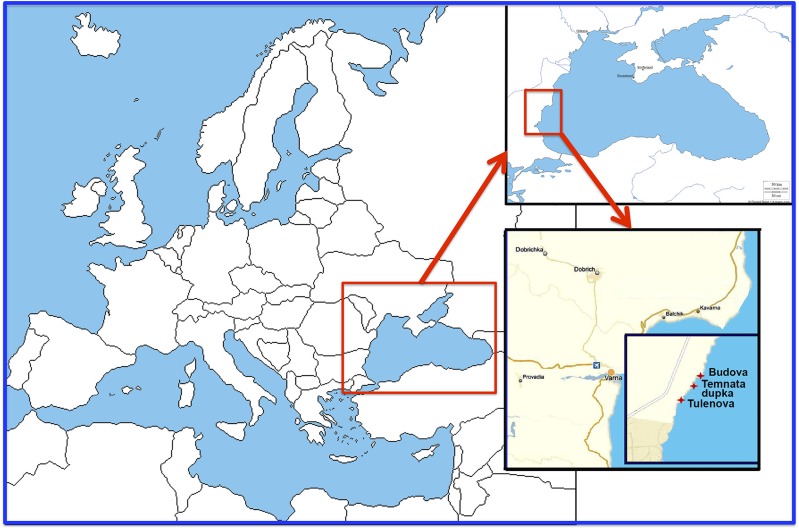
Location of the investigated caves in the North-East Bulgaria (Black Sea).

The research was carried out in 2016 in three semi-submerged marine caves (Budova, Tulenova and Temnata dupka) located in Dobrich province of Bulgaria and at the territory of North-Tyulenovo speleological area of the Eastern-Dobrudzha karst region (Fig. 1). The primary host rock of the caves is thickly layered or massive limestone ranging in color from light grey to white.

### Cave descriptions

Geomorphological descriptions of the caves were conducted using classic geological and hydrobiological methods including three-dimensional mapping (Pronin et al., 2013; Ereskovsky, Kovtun & Pronin, 2016). The coordinates of the cave entrances were determined using a Garmin 12XL GPS. Depths were measured with a diving depth gauge (Mares M2) to an accuracy of 0.1 m. Underwater tunnels, domes and crevices were measured using a reinforced-plastic ruler. Underwater topographic mapping was conducted by the following technique: length and azimuth were assessed between survey pegs placed near the marine cave entrance; then an azimuthal survey was done using an underwater compass and the survey pegs; cross-sections of each cave were drawn using survey pegs. Photographs were taken with a Nikon P6000 digital camera.

### Specimen sampling

Visual exploration and sampling were conducted in 2016 at depths varying from the intertidal zone to 7 m by SCUBA and snorkeling. Sponges were photographed *in situ* with a 5 cm scale bar and an inventory number. Depth and water temperature were recorded by photographing depth gauges at the time of each sampling. Samples were stored in 96% ethanol. The skeletal architecture was studied by light microscopy using thick, polished sections obtained by embedding a fragment of the specimen in Araldite^®^ followed by sectioning with a low speed saw using a diamond wafering blade and wet-ground on polishing discs. Spicule measurements were performed on 25 spicules. All surveys of the caves were digitally recorded using a Sony 3CCD camcorder.

Additional collection of *Dysidea fragilis* (Montagu, 1818) (BsBul16-9-047/GW30179/6 and BsBul16-9-048/GW30180/6) was realized at the South-East of Bulgaria coast: in Agalina cape, rocks, canyon (42°22′43–63″N–27°43′23–83″E) at the depth 4–9 m.

### DNA extraction, PCR and sequencing

For molecular taxonomic support of the morphological results we sequenced two markers frequently used for molecular biodiversity studies of sponges, 5′region of the mitochondrial cytochrome oxidase subunit 1 (in the following referred to as CO1) and the C-Region of the nuclear large ribosomal subunit (in the following referred to as 28S) (see e.g., Erpenbeck et al., 2016). DNA was extracted with the QIAmp mini Kit (Qiagen, Hilden, Germany). The CO1 fragment was amplified using the primers dgLCO1490 (GGT CAA CAA ATC ATA AAG AYA TYG G) and dgHCO2198 (TAA ACT TCAG GGT GAC CAA ARA AYC A) (Meyer & Paulay, 2005). For 28S the primers 28S-C2-fwd (GAA AAG AAC TTT GRA RAG AGA GT) and 28S-D2-rev (TCC GTG TTT CAA GAC GGG) were used (Chombard, Boury-Esnault & Tillier, 1998). The 25 µL PCR mix consisted of 5 µL 5x green Go*Taq*^®^ PCR Buffer (Promega Corp, Madison, WI, USA), 4 µL 25 mM MgCl_2_ (Promega Corp, Madison, WI, USA), 2 µL 10 mM dNTPs, 2 µL BSA (100 µg/ml), 1 µL each primer (5 µM), 7.8 µL water, 0.2 µL Go*Taq*^®^ DNA polymerase (5 u/µl) (Promega Corp, Madison, WI, USA) and 1–2 µL DNA template. The PCR regime comprised an initial denaturation phase of 94 °C for 3 min followed by 35 cycles of 30 s denaturation at 94 °C, 15 s annealing (45 °C for CO1; 51 °C for 28S), 60 s elongation at 72 °C each and a final elongation at 72 °C for 7 min. PCR products were purified with freeze squeeze extractions out of the agarose gels (Tautz & Renz, 1983) before cycle sequencing using the BigDye-Terminator Mix v3.1 (Applied Biosystems). Both strands of the template were sequenced on an ABI 3,730 automated sequencer. Raw sequences were basecalled, trimmed and assembled in CodonCode Aligner v 3.7.1.1 (http://www.codoncode.com). Sequences are deposited in NCBI Genbank under accession numbers MH157894–MH157912, and with location data, specimen photos (including *in situ* photos) and additional morphological information in the form of thin section photos and spicule preparations in the Sponge Barcoding Database (SBD, http://www.spongebarcoding.org, Wörheide & Erpenbeck, 2007), accession numbers SBD #1758–1776.

Phylogenetic analyses were performed after incorporation of all available 28S and CO1 respectively sequences as currently published in NCBI Genbank and aligned in the Sponge Genetree Server (Erpenbeck et al., 2008). Here, secondary structure information as suggested earlier (Erpenbeck et al., 2007) has been implemented where feasible. Phylogenetic reconstructions were performed with RAxML 7.2.8 as implemented in GENEIOUS 8.1.6 (http://www.geneious.com, Kearse et al., 2012) under the GTRCAT model and 100 rapid bootstrap replicates.

### Data collection and taxonomic updating

The scientific literature concerning sponges and benthic invertebrates of the Black Sea was reviewed and all biodiversity data were incorporated into a single annotated Porifera species list along with spatial information. The basis for this was the checklist from previous work (Ereskovsky, Kovtun & Pronin, 2016), which was substantially supplemented.

## Results

All caves investigated are semi-submerged and abrasion karst formed by dense layered light-gray limestone.

### Budova cave

**Figure 2:**
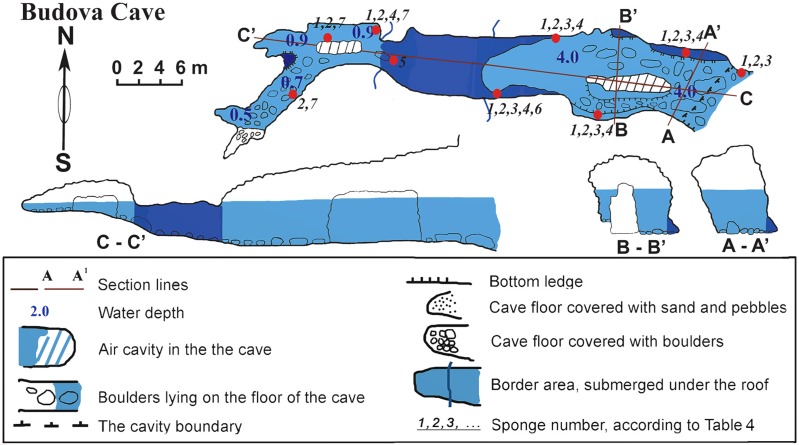
Budova Cave in the North-East Bulgaria, Black Sea.

The cave (PB-105/532) is a semi-submerged cavity with entrance coordinates 43°30′18–8″N and 28°35′35–6″E (Table 1, Fig. 2). Its lower part is flooded with sea-water to a depth of 4.5 m in the entrance decreasing to 1 m towards the end. In the entrance the ceiling is 8 m above the water surface. Maximum width of the cave observed in the entrance is 8.4 m. A big boulder is located at the entrance and a smaller one is in the siphon. The siphon has a sharp rise and further rises gently in the S-W direction above the sea level. The roof of the cave is uneven. The walls have uneven relief with numerous smooth protrusions. The limestone in the walls is corroded. The wall is smoother under water, forming niches along the bottom. The bottom of the cave from the entrance to siphon is almost even and covered with boulders. In the farthest part of the Southern branch, the bottom is rocky. The Northern branch is covered with sand and small well-rounded limestone pebbles. The entire bottom of the cave, except for small areas at the very end, is flooded by seawater.

The cave is hardly accessible when the Eastern and Southern winds blow due to strong wave action. In the inner reaches of the cave wave action is less, allowing for abundant incrustation of aquatic organisms in the wall niches and the small branches of the cave. Illumination is very limited in most parts of the cave, except for the part near the small entrance where the visibility extends to about 10–15 m. However, under the water surface, where diffused light penetrates further, the entrance to the cave can be seen from a distance of 70–80 m in good weather conditions. The room most distant from the entrance is totally dark.

**Table 1:** Main characteristics of investigated underwater caves from North-East Bulgaria.

Caves	Submerged	Semi-submerged	Length (m)	Area (m^2^)	Volume (m^3^)	Depth (m)
Budova	No	Yes	50.2	240.2	936.4	3.5 to 1
Tulenova	No	Yes	83	692	2,150	4 to 1.2
Temnata dupka	No	Yes	73.7	344.5	2,291	3 to 0.5

**Figure 3:**
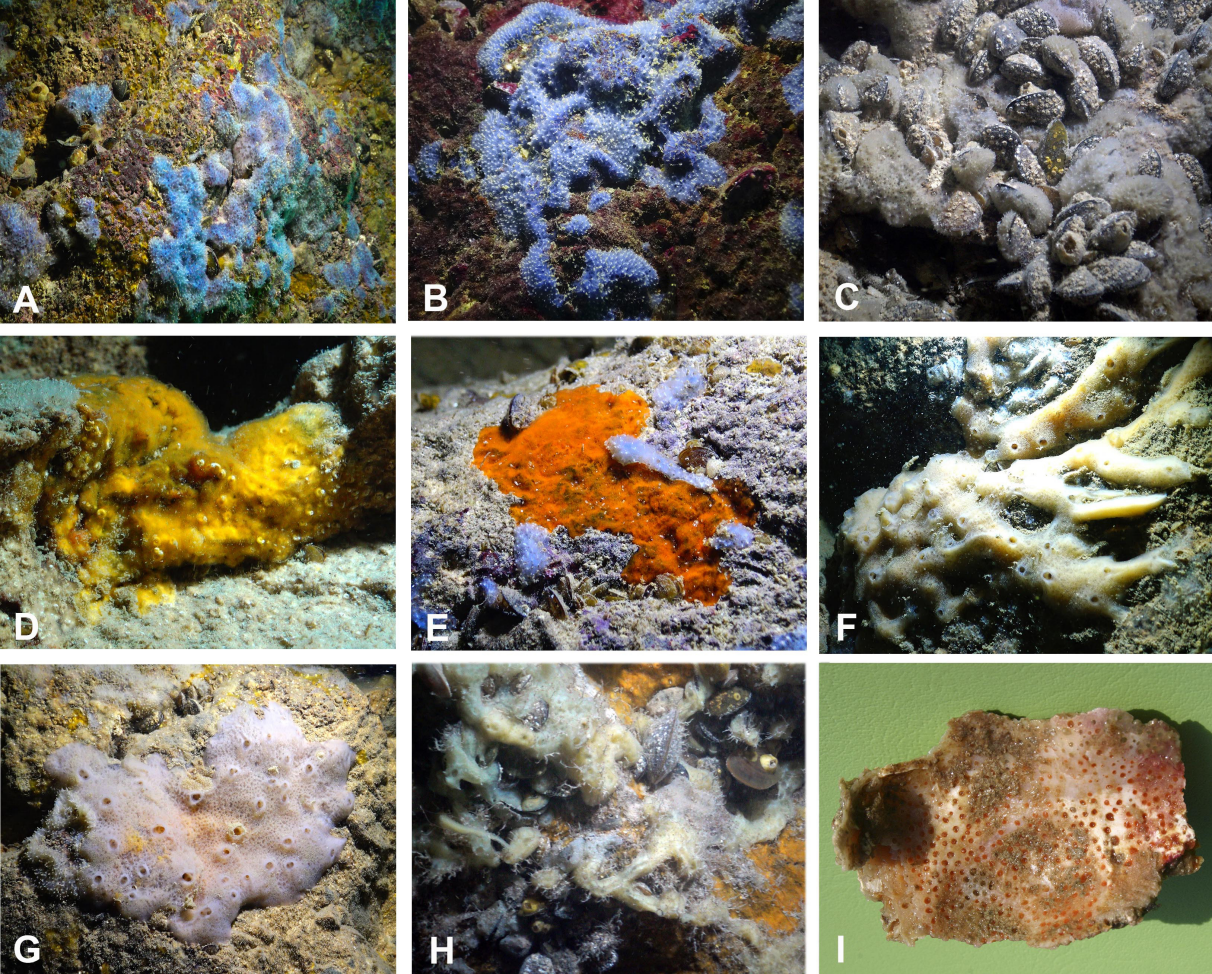
Sponges from the Budova Cave. (A) *Dysidea incrustans* and *Protosuberites denhartogi* at Cave entrance. (B) *Dysidea incrustans in situ* blue morph (cave entrance). (C) *Dysidea incrustans in situ* pale morph in middle part of the Cave. (D) *Protosuberites denhartogi in situ*. (E) *Clathria* (*Microciona*) *cleistochela in situ*; (F) *Halichondria bowerbanki in situ*; (G) *Haliclona* sp. 1 *in situ*; (H) *Haliclona* sp. 2 *in situ*; (I) *Pione* cf. *vastifica. Photographs by Oleg A. Kovtun*.

### Sponges

**Figure 4:**
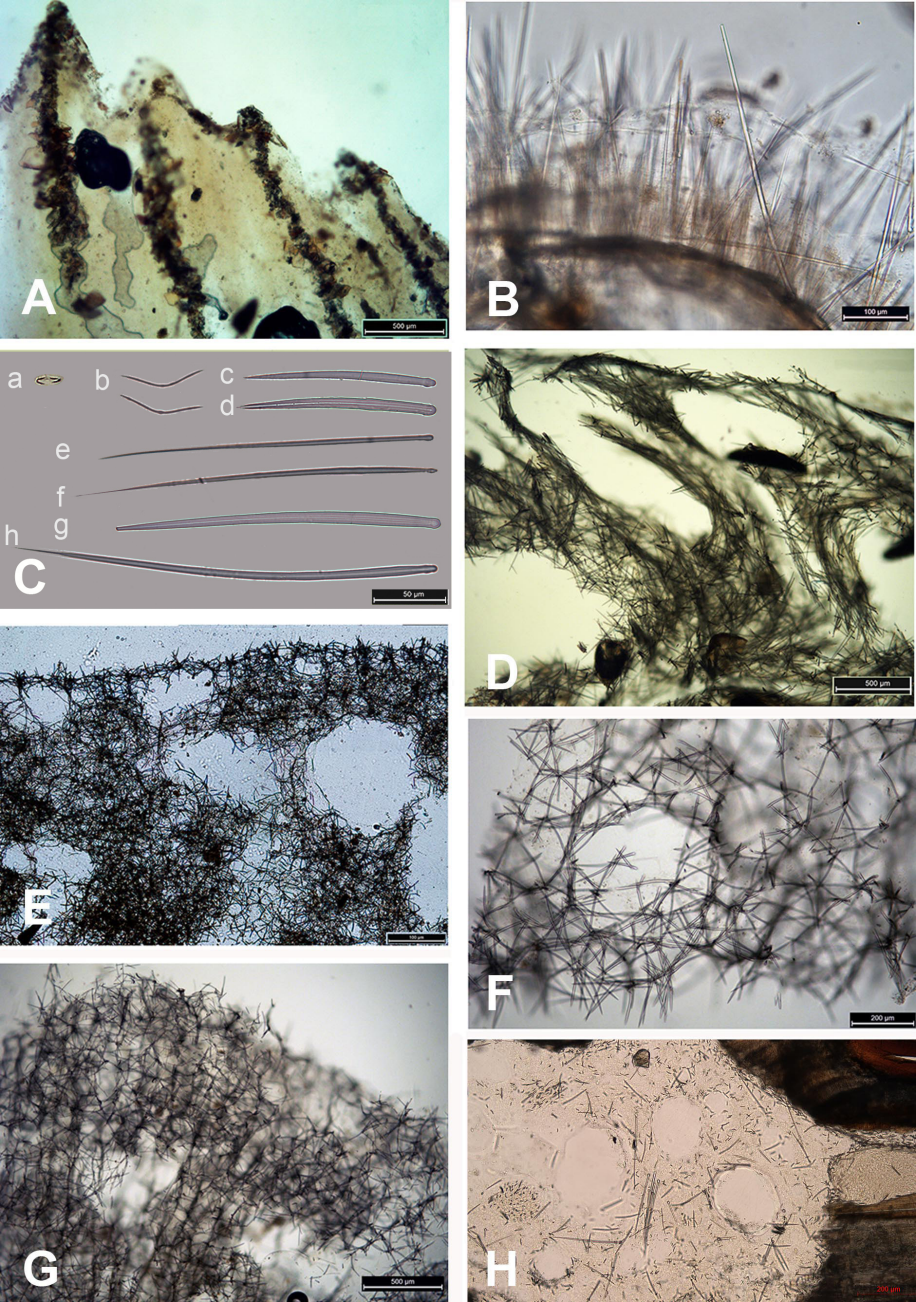
Skeleton and spicular complement of Bulgarian cave sponges. (A) *Dysidea incrustans*. (B) *Protosuberites denhartogi*. (C) Spicules of *Clathria* (*Microciona*) *cleistochela*: a, isochela; b, toxas; c,d, small choanosomal subtylostyles; e,f, large choanosomal subtylostyles; g,h, ectosomal subtylostyles. (D) Skeleton of *Halichondria bowerbanki* and skeleton. (E) Skeleton of *Haliclona* sp. 1. (F) Skeleton of *Haliclona* sp. 2. (G) Skeleton of *Haliclona* sp. 3. (H) Spicules of *Pione* cf. *vastifica. Photographs by Alexander Ereskovsky.*

The species *Dysidea incrustans* (Schmidt, 1862) dominant from the cave entrance as far as section A–A1 (up to 10–15 m deep), on rocky walls and large rocks at the bottom. These sponges are generally bright blue in color with uneven, convex surface, especially towards the illumination. The sponge surface is reticulated and conulose with the conules 1–3 mm high and 3–5 mm apart. Oscula with transparent apical part are scattered. In most cases they are flat crust in shape, with sizes varying from 5 to 15 cm in diameter with a thickness of 1 cm (Figs. 3A, 3B; Video S1). The skeleton network is irregular with meshes of 200–600 µm in diameter formed by ascending primary fibers (70–90 µm in diameter, cored with foreign material), and secondary fibers (5–30 µm in diameter, lacking inclusions) (Fig. 4A). This species also inhabits the large rocks at the bottom of the cave. In the dimly lighted zones of the cave between sections A–A1 and B–B1, *D*. *incrustans* occurs less frequently and can display a flattened habitus and pale violet color.

A similar distribution pattern is exhibited by *Protosuberites denhartogi* van Soest, de Kluijver, 2003, an encrusting and mustard yellow in color sponge (Figs. 3A, 3D; Video S1), which covers not only the rocky surface but harbors colonies of different barnacles species. The sponge surface is even, smooth and slightly hispid. Consistency is compact. Ectosomal skeleton is lacking special architecture, with short tylostyles, oriented parallel to the surface. At the surface the spicule bundles usually support projecting spicule tufts. The choanosomal skeleton consists of tylostyles arranged perpendicularly to the substrate, with the heads directed to the substrate (Fig. 4B). In thicker specimens, basal spicules are overlain by spicule bundles that anastomose and may run either perpendicular or parallel to the substrate. Overall spicule density is high. The megascleres are tylostyles, considerably variable but overlapping in size, without distinct size categories, 110–258.7–456 × 4–6.3–11 µm. Microscleres are absent.

In this part of the cave the surface of *P. denhartogi* can reach more than 40 cm^2^ with not more than 1-3 mm thickness. In the middle part of the cave the surface of *P. denhartogi* is reduced to 2–5 cm^2^.

Another common species in the entrance area is *Clathria* (*Microciona*) *cleistochela* (Topsent, 1925) as a thin, orange crust up to 20–30 cm in diameter (Fig. 3D; Video S1). Its ectosomal skeleton is composed of monactinal auxiliary spicules in one or two categories forming sparse, paratangential structures. The choanosomal skeletal tracts are usually enclosed within spongin fibers and embedded megascleres and are erect on basal layer. The skeleton of the sponge consists of megascleres in form of ectosomal subtylostyles (175–320 µm long) and choanosomal subtylostyles of two-dimensional categories: large (up to 475 µm long) and small (up to 175 µm long); and microscleres in form of numerous isochelae (13–15 µm in length) and toxas of the same size category (about 150 µm in length) (Fig. 4C). In the middle part of the cave the surface of this species is reduced to 3–7 cm in diameter, where it is covered by numerous *Spirorbis* polychaetes.

In the middle part of the cave beyond section B–B1, where the marked narrowing of the entrance leads to a drastic reduction of incoming light, the bottom part of the wall is encrusted by the green-gray species *Halichondria bowerbanki* Burton, 1930 (Fig. 3E; Video S1). This sponge is moderately firm and compressible in consistence. The oscules are highly variable in size and position. The ectosomal skeleton is tangential with spicules arranged disorderly or in tight bundles, leaving open spaces between spicule bundles. The choanosomal skeleton is largely confused. It consists of oxea tracts running toward the surface and anastomose at intervals (Fig. 4D). These tracts contain between 6 and 12 spicules. The spicules are oxea megascleres only: long, thin, straight or gently curved at the midpoint and varying considerably in size from 135 to 390 µm long and 3.7 to 13.1 µm wide.

In the same cave zone there is a rare *Haliclona* (referred to as *Haliclona* sp. 1 hereafter), a flat sponge of grey-blue color (Fig. 3F; Video S1) with diameters of 7–10 cm, and with small outstretched oscular chimneys of about 5–8 mm. The sponge surface is even and smooth, slightly hispid due to projecting spicules. The ectosomal skeleton is a rather regular, tangential, unispicular, with isotropic reticulation; the choanosomal skeleton is a regular, unispicular, paucispicular reticulation (Fig. 4E). The spicules are exclusively oxeas of uniform shape and size: 64–100 × 2–4 µm. No microscleres are present.

The inner, dark parts of the cave are inhabited by small oval and pale blue *D. incrustans* (Fig. 3C), and a different, large encrusting *Haliclona* species (referred to as *Haliclona* sp. 2). *Haliclona* sp. 2 has ivory color with long, fine outgrowths at the surface (Fig. 3G; Video S1). The sponge surface is hispid due to projecting spicules. The ectosomal and choanosomal skeletons are tangential. Primary multispicular and secondary unispicular tracts have a regular, triangular reticulation (Fig. 4F). The spicules constitute exclusively megascleres oxeas of uniform shape and size: 150–190 × 6.5–9 µm. In this dark part of the cave numerous small crusts (1–3 cm in diameter) of *P. denhartogi* are present. Finally in the terminal and totally obscured post-syphon zone small individuals of *P. denhartogi* and big specimens of *Haliclona* sp. 2 with outgrowths were observed.

The walls of the cave have been perforated by the boring sponges *Pione* cf. *vastifica* (Hancock, 1849) (Fig. 3H). The choanosomal skeleton of *P.* cf. *vastifica* consists of randomly arranged microspined oxeas and scattered tylostyles and oxeas, sometimes arranged in bundles. The superficial skeleton is organized by spirasters parallel arranged to the surface (Fig. 4H). The skeleton of the papillae is composed by tylostyles (160–340 µm long and 3–9 µm in diameter) and spiraster microscleres. The spirasters are 8–25 µm long and 1–2 µm in diameter, straight or with a bent shaft and truncated ends and small spines. Additional microscleres are microspined oxeas with slightly bent shaft of 60–140 µm length and 2–6 µm in diameter.

The vertical distribution pattern of sponges in the post-syphon area is typical for an open-water area: the largest number of sponges is found in the lower and middle parts of the cliffs, while the upper 2–3 m of rock is almost free of sponges due to the strong hydrodynamic effects of storm activity in this zone.

### Tulenova cave

**Figure 5:**
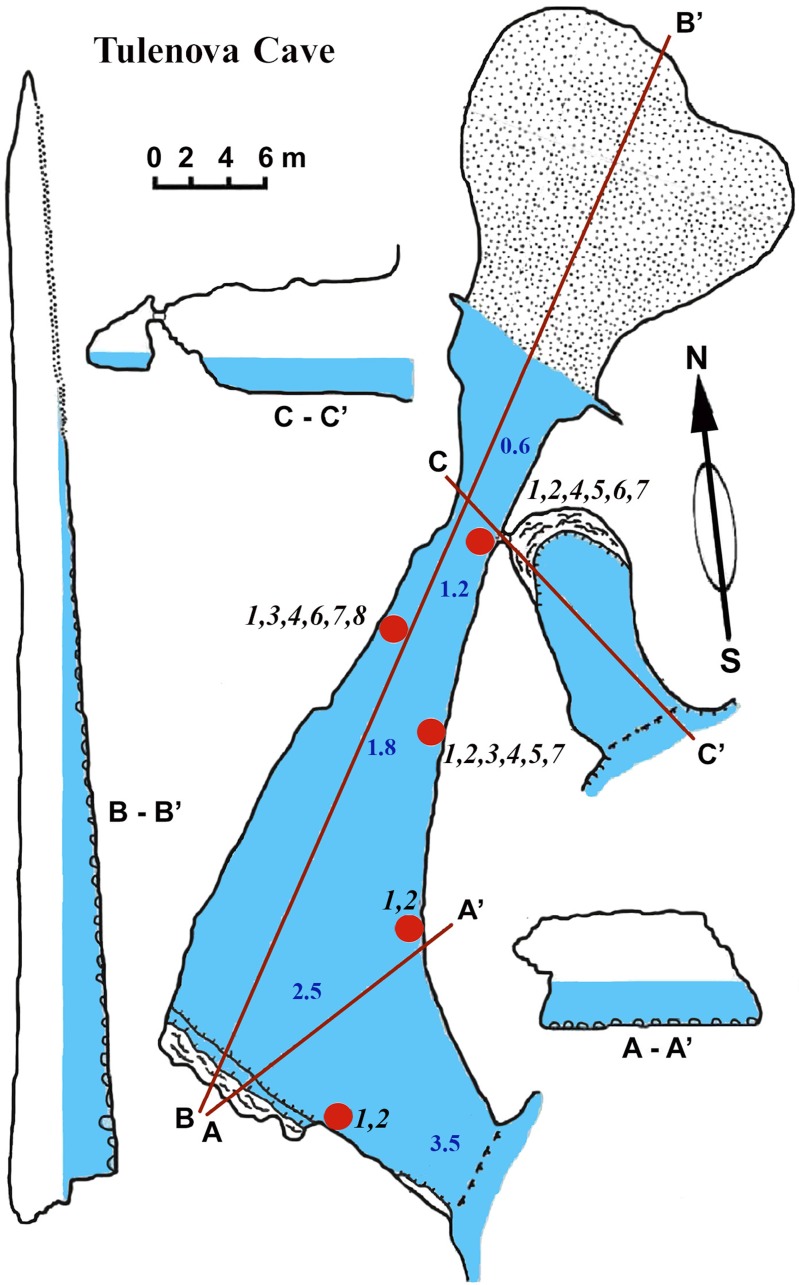
Tulenova Cave in the North-East Bulgaria, Black Sea.

This cave (PB-105/550) has the principal entrance coordinates 43°29′40–52″N and 28°35′05–58″E (Table 1, Fig. 5). The Southern part of the cave is situated from the entrance to A–A1. Then the main part turns almost 90°. Large caverns and niches, about 0.5 m high, are present in the Southern part of the cave. The surfaces of the walls are uneven, with an angular, flat roof. On the South side at a depth of about 0.5 m a smooth rocky ledge is present. The wall between the ledge and the flat roof of the cave is very uneven, sloping, with large niches and ledges. The parts North of A–A1, on which the mainsail narrows, and the connection to the Eastern part are similar in nature. After the restriction on C the cave is expanding rapidly and reaches its maximum width of 18 m with rounded and smooth outlines. Its bottom is covered with sand and gently rises above sea level. The water depth at the input reaches 3.5 m. With growing distance from the cave entrance water depth gradually decreases. Most of the bottom of the cave is flooded. Almost the entire bottom of the cave halts limestone boulders. Sometimes there are small areas of rock bottom—smooth ledges located at a depth of 0.5 m. On the far side of the cave there is a large sand and pebble beach with detritus and a height of 1 m.

### Sponges

**Figure 6:**
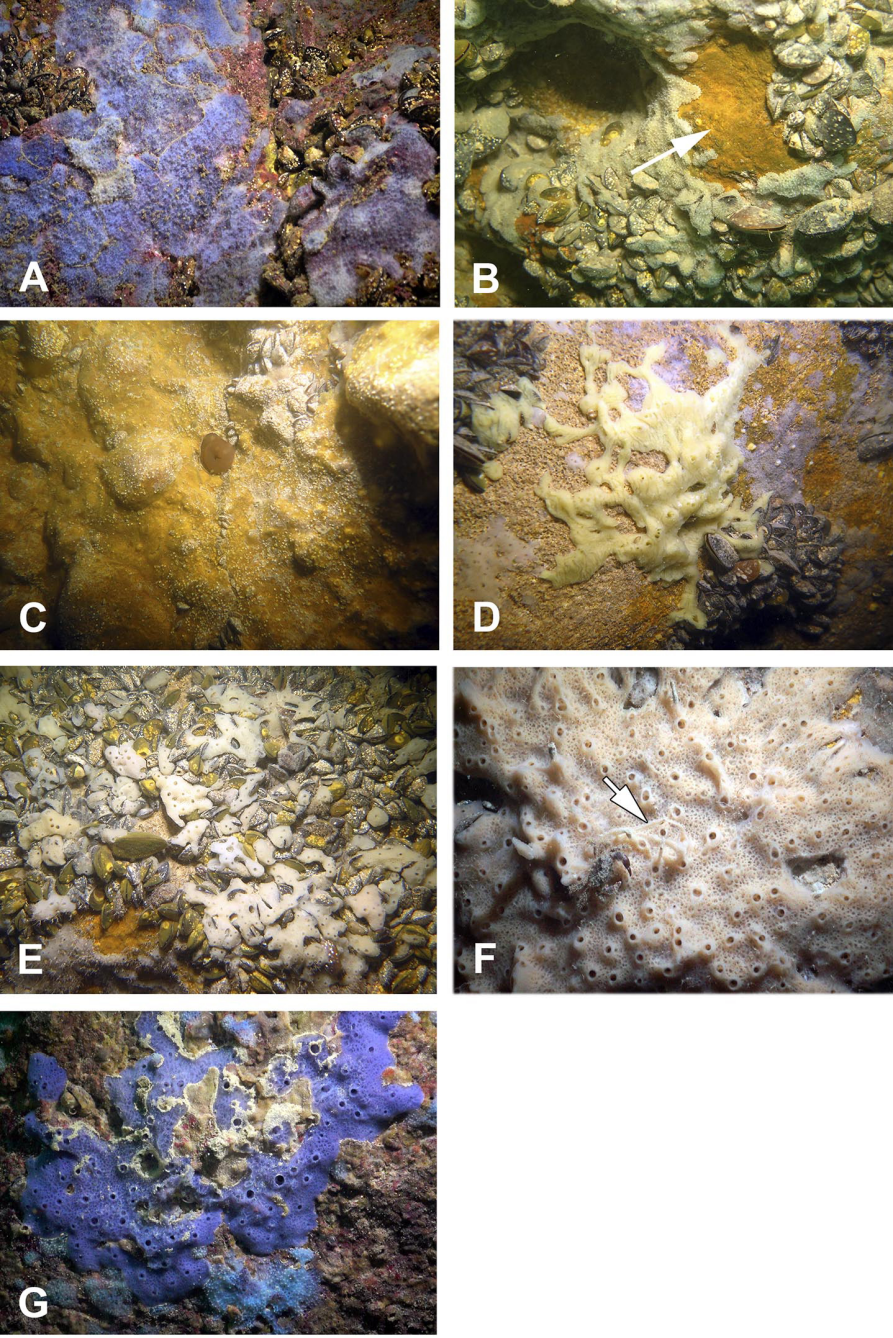
Sponges from the Tulenova Cave. (A) *Dysidea incrustans* *in situ* blue morph at Cave entrance. (B) *Clathria* (*Microciona*) *cleistochela in situ* (arrow); (C) *Protosuberites denhartogi in situ*. (D) *Halichondria bowerbanki in situ*; (E) *Haliclona* sp. 1 *in situ*; (F) *Haliclona* sp. 2 with outgrowths (arrow) *in situ*; (G) *Haliclona* sp. 3 *in situ. Photographs by Oleg A. Kovtun*.

The sponge distribution in the entrance zone of the cave is different from that of the rocks outside the cave entrance. The entrance of the cave is completely exposed to waves and light, so the cave *per se* begins with section A–A1, where the distribution of sponges is typical of dark areas. At the entrance the small pale-blue sponge *Dysidea incrustans* (Fig. 6A; Video S2) is reduced in number. However, *Protosuberites denhartogi* (Fig. 6C; Video S2) constitute the dominant species in this part of the cave with a projective cover about 60–70%. The sponges overgrow *Spirorbis* polychaetes, barnacles, and mussels. Behind section A–A1, the Eastern side of the cave differs from the Western in the abundance of epibionts. The Eastern wall of the cave, rich in different niches and cavities, is more densely populated by sponges. Up to section C–C1 *P. denhartogi* is the dominant sponge species, but only in the upper and middle part of the cave. From the middle to the depth part, a flat white-gray *Haliclona* sp. 2 (Fig. 6F; Video S2) is more abundant in niches and recesses as well as on the mussel shells. In relatively hydrological calm zones some individuals of *Haliclona* sp. 2 have long outgrowths beginning from the peripheral part of oscular tubes. *D. incrustans* is quite numerous in the deep zone of the cave; the individuals of this species have rater small dimensions and pale-blue color and inhabit the shells of numerous mussels. Specimens of *Haliclona* sp. 1 are found in the same zone. This species has small size (2–5 cm), and white color (Fig. 6E; Video S2). There is also a third *Haliclona* species, *Haliclona* sp. 3, with a characteristic violet-blue colour (Fig. 4G; Video S2). The sponge surface is slightly hispid due to projecting ends of choanosomal tracts in varied positions. The choanosomal skeleton is regular, consists of primary multispicular (3–4 spicules) and secondary unispicular tracts form triangular meshes (Fig. 6G). Spicules include only slender and fusiform oxeas: 120–145 × 3.7–5 µm and no microscleres.

The rare species *Halichondria bowerbanki* (Fig. 6D; Video S2) is also found in this zone. This sponge has distinct dermal membrane and chains of oscula at the surface. In addition the encrusting sponge *Clathria cleistochela* (Fig. 6B; Video S2) is found on vertical walls side. This species species has small size and is rare in Tulenova Cave. The rocks in the niches are also perforated by the boring sponge *Pione* cf. *vastifica*.

**Figure 7:**
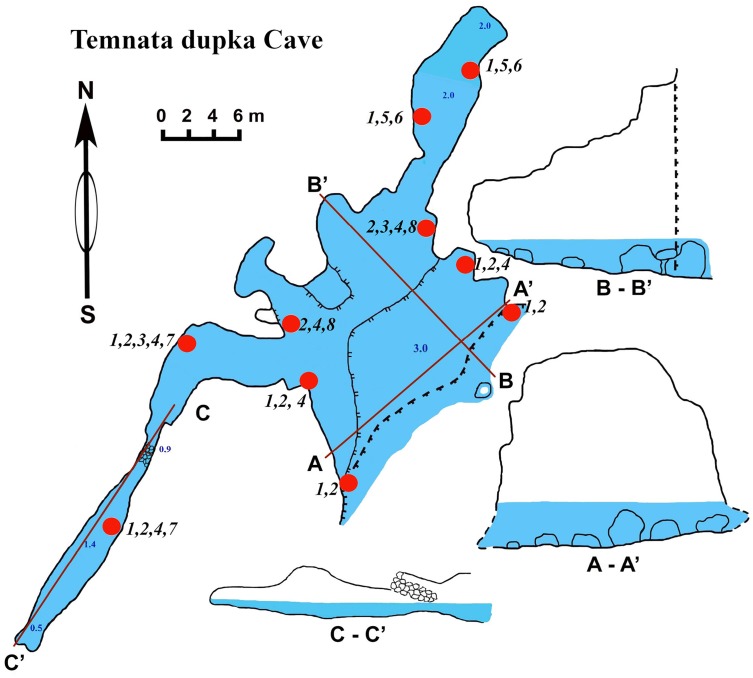
Temnata dupka Cave in the North-East Bulgaria, Black Sea.

### Temnata dupka cave

The cave (PB-105/496) entrance is located at 43°30′15″N, and 28°35′32–16″E (Table 1, Fig. 7). This cave is a large wave-cut niche with trapezoidal shape. On the far side of the cave, there are several branches/niches. Two of them, in the terminal wall, have small dimensions. The other two branches, extending in the North-East and South-West directions, are comparatively large—the South-West branch has a length of about 30 m. The bottom falls slightly, but then is raised again to a dead end. The height from the water surface to the arch of the dome is 2.5 m with water depths of 1.5 m. The North-East branch length of 16 m is very different. Its water depth at the end of branch reaches 3.5 m. The cross section is rectangular only at the beginning of the branch, and at the middle part it is close to triangular. The surfaces of the walls are more angular, although projections are smoothed. The roof is uneven. The maximum height of the cave at the entrance is 15 m (of which 3 m are flooded). Basically, the cave height comprises about 7–9 m and a maximum width of 18.5 m. The bottom of the main part of the cave is almost flat, slightly rising in the end of cave and covered with boulders, pebbles and sand. The entire bottom is flooded. The water depth in the entrance parts is up to 3 m, in the North-Eastern branch about 2 m, in the South-West branch maximum depth is 1.4 m, in the end 0.5 m. During storms the access to the cave is difficult.

### Sponges

**Figure 8:**
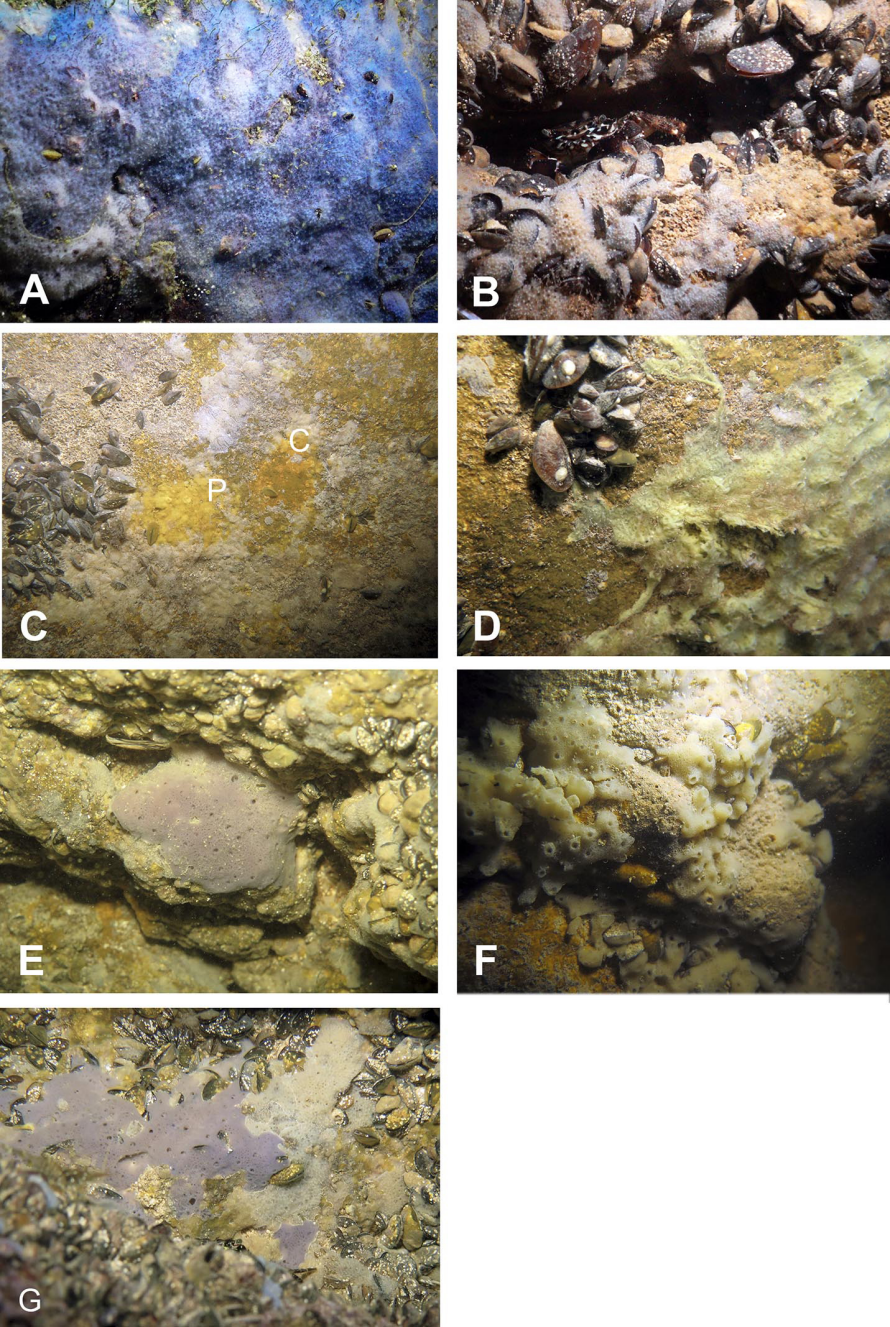
Sponges from the Temnata dupka Cave. (A) *Dysidea incrustans* *in situ* blue morph at Cave entrance. (B) *Dysidea incrustans in situ* pale morph middle part of the Cave. (C) *Clathria* (*Microciona*) *cleistochela* (C) and *Protosuberites denhartogi* (P) *in situ*. (D) *Halichondria bowerbanki in situ*; (E) *Haliclona* sp. 1 *in situ*; (F) *Haliclona* sp. 2 *in situ*; (G) *Haliclona* sp. 3 *in situ. Photographs by Oleg A. Kovtun*.

Numerous blue colored *Dysidea incrustans* specimens inhabit the entrance area of the cave, as well as the surrounding rocks (Fig. 8A: Video S3). Similarly to other caves, *Protosuberites denhartogi* is one of dominant species in this area with a projective cover about 30–40% (Fig. 8C; Video S3). In the middle part of the cave individuals of *D. incrustans* are small in the beginning, very thin, of whitish color, and very numerous (Fig. 8B; Video S3). The number of this species decreases in the North-Eastern outgrowth of the cave. Here, they often grow on mussels. In this area there are many big individuals of *Haliclona* sp. 1 with violet-whitish color and relatively big oscula. The sponges have various shapes from oval to irregular (dimension ranging from 3 × 5 cm to 5 × 10 cm) (Fig. 8E; Video S3). In the North-Eastern outgrowth of the cave large, encrusting *Halichondria bowerbanki* specimens are found (Fig. 8D; Video S3), while on vertical walls in the middle part, several *P. denhartogi* and *Clathria cleistochela* (Fig. 8C; Video S3) specimens can be observed. Encrusting *Haliclona* sp. 3 of blue-gray color with prominent oscular chimneys up to 8 mm (Fig. 8G; Video S3) was rarely found. In the Western outgrowth of the cave there are many small blue *D. incrustans*, rare small *P. denhartogi* and encrusting *Haliclona* sp. 2 of ivory colour. *Pione* cf. *vastifica* perforates the surface of rocks and the shells of old mollusks.

### Molecular results

**Figure 9:**
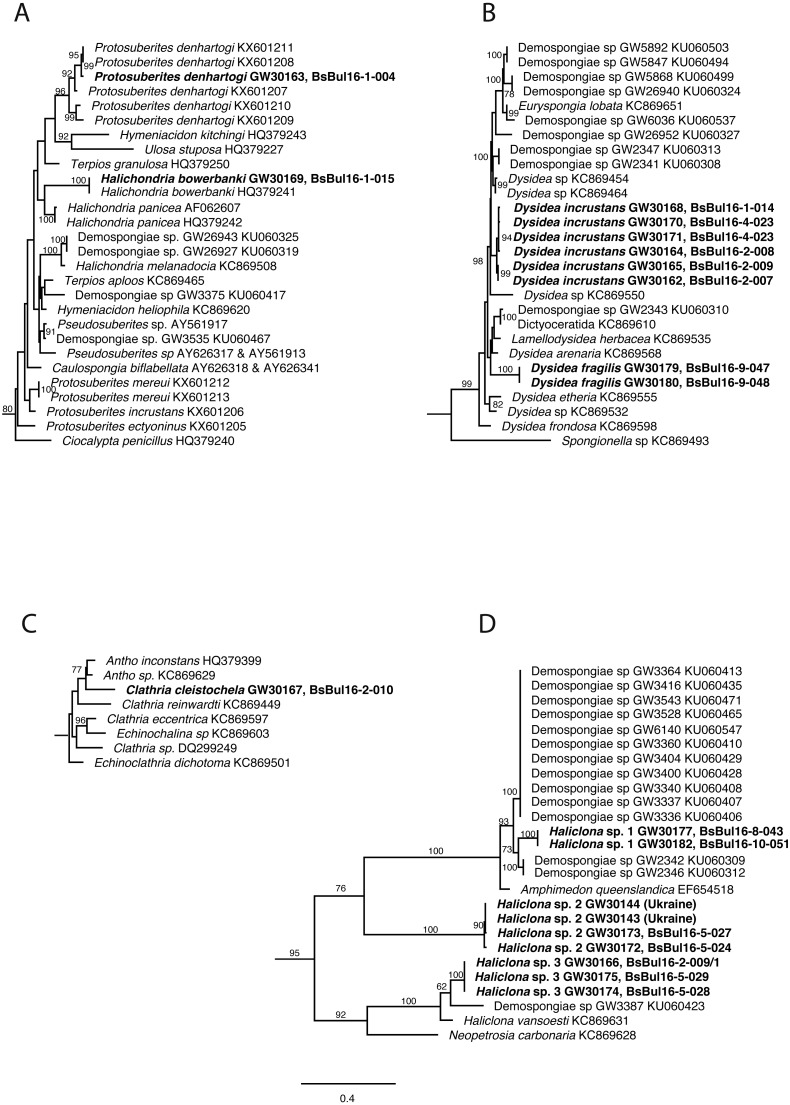
Excerpts from the Maximum Likelihood phylogenetic reconstructions of in total 1,108 sequences in 28S rDNA (C-region) for species collected in the Bulgarian caves. (A) *Halichondria bowerbanki* and *Protosuberites denhartogi*; (B) *Dysidea* spp.; (C) *Clathria cleistochela*; (D) *Dysidea* spp. Numbers on branches indicate bootstrap support >70. Taxon names in bold are specimens newly sequenced and referred to in this work. Numbers following taxon names indicate either Genbank accession numbers or collection numbers of the Bavarian State collections for Paleontology and Geology ([SNSB-BSPG.]GWxxxxx) plus field collection numbers (BsBulxxxx). The scale bar indicates substitutions per site for all figures.

DNA of the relevant sponge species has successfully been extracted, amplified and 28S and CO1 sequenced. The final trees comprised 1,108 (28S) and 2,531 taxa (CO1) respectively. Relevant excerpts of the phylogenetic trees are displayed in Figs. 9A–9D. The samples fall into the four orders Haplosclerida, Dictyoceratida (Dysideidae), Suberitida, and Poecilosclerida (Microcionidae) as expected. *Protosuberites denhartogi* results in a distinct monophyletic clade with other conspecifics. *Halichondria bowerbanki* is identical to a previously published *H. bowerbanki* from the North Sea (HQ379241). For *Dysidea* spp., *Haliclona* spp. and *Clathria cleistochela,* no conspecific, or identical 28S counterpart was found.

## Discussion

### Geological and morphological features of the caves

In spite of a high number of underwater caves in northern and western parts of the Black Sea (Pronin, 2010; Pronin, 2011) biological investigations were conducted up to now only for the Bulgarian coast and the Crimean Tarkhankut Peninsula (Kovtun & Makarov, 2011; Petrjachev & Kovtun, 2011; Vorobyova et al., 2012; Ereskovsky, Kovtun & Pronin, 2016); this work). These two areas are similar in their geological structure, despite their separation of about 400 km. The morphological structure of their coastlines is also similar, displaying steep banks of 15-40 m height, vertically extending into the sea. Below sea level we find cliffs of up to 6 m before the bottom becomes shallow.

The Bulgarian sites are nearly straight, stretching from North to South, and the entrances to the caves are exposed to the East (Pronin et al., 2013). Due to the straightness of the banks, these areas very often undergo strong wave disturbances. The coastline of the Tarkhankut is oriented East-West, and the entrances to the caves are exposed to the South (Kovtun & Pronin, 2011a; Kovtun & Pronin, 2011b; Pronin, 2011).

The sea coastal cliffs in the Bulgarian and Crimean regions are composed of medium-Sarmatian limestones with identical structures of its layers. Their largest caves, including those mentioned in this paper, are formed in the lower part of the coastal cliffs in dense monolithic or thick-layered limestone. Consequently, the shallow-water semi-submerged caves located on these two shores are morphologically similar.

The shallow semi-submerged caves of Crimea are different in their morphometric parameters (Ereskovsky, Kovtun & Pronin, 2016). Their length differ by factor 10 and the area by factor 29 (from 9 m in the cave PK-324, to 101 m in the cave “Love”) (Table 2). The Bulgarian caves studied in this work are more homogeneous, their differences are smaller, their length differ by factor 1.7, and the area differs in 2.7 times (caves of Budova and Tulenova) (Table 2).

**Table 2:** Comparison of shallow-water semi-submerged caves of Tarkhankut (Crimea) and North-East Bulgaria.

Crimea	Length (m)	Depth (m)	Area (m^2^)	Width of the enter (m)	Sponge species number	Bulgaria	Length (m)	Depth (m)	Area (m^2^)	Width of the enter (m)	Sponge species number
Love	101	4–1.2	1,168	27	7	Tulenova	83	4–1.2	692	19	8
PK-356	24	3–0.8	130	7	4	Temnata dupka	74	3–0.5	345	19	8
PK-324	9	2.5–1	40	4	4	Budova	50	3.5–1	240	8	7

### Sponge fauna

The analysis of the sponge material reveals that the shallow-water caves in Bulgaria differ in species diversity from caves in Crimea (caves: Love, PK 356, PK 324) (Ereskovsky, Kovtun & Pronin, 2016). In particular, *Geodia stellosa* Czerniavsky, 1880 ordinary inhabitant of the caves of Tarkhankut is absent from the Bulgarian caves, while *Halichondria bowerbanki* often found in Bulgaria, was not reported from caves of Crimea (Table 3). At the same time, the caves of Bulgaria are more diverse in *Haliclona* species: seven morphotypes, representing at least three molecularly distinguishable species, are found (Fig. 9). Our observations show that some species in Bulgarian caves reach larger size (e.g., *Halichondria bowerbanki*, *Dysidea incrustans*, *Protosuberites denhartogi*).

**Table 3:** Taxonomical composition and distribution of Black Sea sponge fauna. Numbers refer to the references given at the bottom of this table.

Sponge taxon	Sponge distribution in Black Sea							
	Romania	Bulgaria	Turkey	Georgia	Russia	Ukraine NW	Crimea	Crimea caves
**Class Calcarea** **Subclass Calcaronea** **Order Leucosolenida** Family Sycettidae								
*Sycon ciliatum* (Fabricius, 1780)	1, 16	1	2		3		4, 26	
*Sycon setosum* Schmidt, 1862	1		2				5	
*Sycon tuba* Lendenfeld, 1891			2					
*Sycon* cf. *vigilans* Sarà & Gaino, 1971							28	Unpubl
**Class Demospongiae** **Order Tetractinellida** Family Geodiidae								
*Geodia stellosa* Czerniavsky, 1880		6	6		6		5, 9	
*Stelletta* sp*.*								29
**Order Suberitida** Family Suberitidae								
*Suberites domuncula* (Olivi, 1792)	1	1	2	9		8, 9	9, 10	
*Suberites carnosus* (Johnston, 1842)	1, 6	6	2		6	4	4	
*Protosuberites brevispinus* (de Laubenfels, 1951)		1, 25						
*Protosuberites denhartogi* van Soest & de Kluijver, 2003		27^a^						
*Protosuberites mereui* Manconi, 2016						Unpubl	Unpubl	Unpubl
*Protosuberites prototypus* Swartschewsky, 1905		1, 6		9	8		8, 10	7
Family Halichondriidae								
*Halichondria panicea* (Pallas, 1766)	1	1, 22	6		9	8	8	
*Halichondria bowerbanki* Burton, 1930		27^a^						
*Halichondria* (*Halichondria*) *foraminosa* (Czerniavsky, 1880)							9	
*Halichondria* (*Halichondria*) *longispicula* (Czerniavsky, 1880)							9	
*Halichondria* (*Halichondria*) *pontica* (Czerniavsky, 1880)					9	9	10	
*Hymeniacidon luxurians* (Lieberkühn, 1859)							10	
*Hymeniacidon perlevis* (Montagu, 1818)		18, 22	4		4	4	8	
**Order Clionaida**								
Family Clionaidae								
*Pione* cf. *vastifica* (Hancock, 1849)	1, 6	1, 27^a^			9	11	9, 10	7
*Cliona lobata* Hancock, 1849	1, 6							
**Order Poecilosclerida** Family Mycalidae								
*Mycale* (*Aegogropila*) *contarenii* (Lieberkühn, 1859)		1		8	9	8	4, 9	
*Mycale* (*Aegogropila*) *dubia* (Czerniavsky, 1880)							9	
*Mycale* (*Aegogropila*) *syrinx* (Schmidt, 1862)	16	1			3	4	4, 26	
*Mycale stepanovii* (Czerniavsky, 1880)							9	
*Mycale lobimana* (Czerniavsky, 1880)							9	
*Mycale jophon* (Swartschewsky, 1905)							10	
*Mycale muscoides* (Czerniavsky, 1880)						8	8, 9, 10	
*Mycale* (*Mycale*) *simplex* (Czerniavsky, 1880)							9	
Family Myxillidae								
*Myxilla* (*Myxilla*) *swartschewskii* Burton, 1930	1			4		6	4	
Family Tedaniidae								
*Tedania* (*Tedania*) *anhelans* (Olivi, 1792)	1	1	2				9	
Family Coelosphaeridae								
*Lissodendoryx* (*Lissodendoryx*) *variisclera* (Swartschewsky, 1905)	1, 6	24				6	8, 9	
Family Crellidae								
*Crella* (*Crella*) *elegans* (Schmidt, 1862)							10, 26	
*Crella* (*Yvesia*) *gracilis* (Alander, 1942)		1		4		6	8	
Family Hymedesmiidae								
*Hymedesmia* (*Stylopus*) *coriacea* (Fristedt, 1885)					8	8	8, 10	
*Hymedesmia* (*Hymedesmia*) *pansa* Bowerbank, 1882			17					
*Hymedesmia* (*Hymedesmia*) *veneta* (Schmidt, 1862)							10	
Family Microcionidae								
*Clathria* (*Microciona*) *cleistochela* (Topsent, 1925)	12	1, 27^a^						13
*Antho* (*Antho*) *involvens* (Schmidt, 1864)					9			
**Order Haplosclerida** Family Chalinidae								
*Chalinula limbata* (Montagu, 1818)		1						
*Chalinula renieroides* Schmidt, 1868			17					
*Haliclona alba* (Schmidt, 1862)		1	2		9		9	
*Haliclona albapontica* (Czerniavsky, 1880)							9	
*Haliclona boutschinskii* (Kudelin, 1910)						14		
*Haliclona cribrosa* (Czerniavsky, 1880)							9	
*Haliclona curiosa* (Swartschewsky, 1905)							10	
*Haliclona cylindrigera* (Czerniavsky, 1880)							9	
*Haliclona densa* (Lendenfeld, 1887)	1, 6	1, 6					10	
*Haliclona flavescens* (Topsent, 1893)	1	1, 19, 25				4	8	7
*Haliclona foraminosa* (Czerniavsky, 1880)					9		9	
*Haliclona* (*Halichoclona*) *fulva* (Topsent, 1893)			17					
*Haliclona gracilis* (Miklucho-Maclay, 1870)	1, 6				3	6	8	
*Haliclona inflata* var*. taurica* (Czerniavsky, 1880)							9, 10	
*Haliclona informis* var. *taurica* (Czerniavsky, 1880)		1			9		9, 10, 26	
*Haliclona irregularis* (Czerniavsky, 1880)					9		10	
*Haliclona odessana* (Kudelin, 1910)						14		
*Haliclona palmata* (sensu Lieberkühn, 1859)					9		9, 10	
*Haliclona nigricans* (Czerniavsky, 1880)							9	
*Haliclona pontica* (Czerniavsky, 1880)						8	9	
*Haliclona* (*Rhizoniera*) *rosea* (Bowerbank, 1866)			17					
*Haliclona schmidti* (Czerniavsky, 1880)							9	
*Haliclona transitans* (Czerniavsky, 1880)							9	
*Haliclona tubulifera* (Swartschewsky, 1905)		1					10	
*Haliclona* (*Gellius*) *angulata* (Bowerbank, 1866)		6			6	15	15	
*Haliclona* (*Haliclona*) *simulans* (Johnston, 1842)		1				6	26	
*Haliclona* (*Reniera*) *aquaeductus* var*. taurica* (Czerniavsky, 1880)	1, 6	1, 6	2			6	10	
*Haliclona* (*Reniera*) *cinerea* (Grant, 1826)	1, 6	1, 6			6	6	5	
*Haliclona* (*Reniera*) *cratera* (Schmidt, 1862)		6	2					
*Haliclona* (*Rhizoniera*) *grossa* (Schmidt, 1864)		6	2				10	
*Haliclona* (*Soestella*) *implexa* (Schmidt, 1868)	1, 6	1				11	5	
*Haliclona* sp.1		27^a^						
*Haliclona* sp.2		27^a^						
*Haliclona* sp.3		27^a^						
*Haliclona* sp.4(2)								7
Family Petrosiidae								
*Petrosia* (*Petrosia*) *clavata* (Esper, 1794)							26	
*Petrosia coriacea* Swartschewsky, 1905							10	
*Petrosia ficiformis* (Poiret, 1789)	1	1			11	11	11	
*Petrosia* (*Petrosia*) *intermedia* (Czerniavsky, 1880)					9		9, 10	
Family Phloeodictyidae								
*Oceanapia ascidia* (Schmidt, 1870)					6	6	5, 10	
**Order Dictyoceratida** Family Dysideidae								
*Dysidea fragilis* (Montagu, 1818)	1,6	1, 20, 21, 23, 25	2			6	8, 26	7
*Dysidea elegans* var. *pontica* (Czerniavsky, 1880)					9	6	4	
*Dysidea avara* (Schmidt, 1862)					9		9	
*Dysidea incrustans* (Schmidt, 1862)		27^a^				9		9
*Dysidea pallescens* (Schmidt, 1862)							9	
Family Irciniidae								
*Ircinia variabilis* (Schmidt, 1862)			17					
Family Spongiidae								
*Spongia* (*Spongia*) *officinalis* Linnaeus, 1759		6	2					
**Order Chondrillida** Family Halisarcidae								
*Halisarca dujardini* Johnston, 1842			2			9	9	

**Notes.**

1, Gomoiu & Skolka, 1998; 2, Topaloğlu & Evcen, 2014; 3, Terentiev, 1998; 4, Kaminskaya, 1968; 5, Kiseleva & Kostenko, 2004; 6, Bačescu, Muller & Gomoiu, 1971; 7, Ereskovsky, Kovtun & Pronin, 2016; 8, Kaminskaya, 1961; 9, Czerniavsky, 1880; 10, Swartschewsky, 1905; 11, Kaminskaya, 1967; 12, Skolka & Gomoiu, 2004; 13, Ereskovsky & Kovtun, 2013; 14, Kudelin, 1910; 15, Kaminskaya, 1966; 16, Begun, Teacă & Gomoiu, 2010; 17, Evcen et al., 2016; 18, Christie et al., 1994; 19, Elenkov, Popov & Andreev, 1999; 20, Christie et al., 1992; 21, De Rosa et al., 2000; 22, Elenkov, Popov & Andreev, 1999; 23, Elenkov et al., 1994; 24, Elenkov et al., 1996; 25, Laubenfels, 1951; 26, Galtsoff, 1923; 27, this work; 28, F Azevedo, pers. comm., 2017; 29, P Cardenas, pers. comm., 2017.

a Sponges from the caves of Bulgaria.

For all three investigated caves sponge distribution is similar in the entrance zone. The dominant species here are *D. incrustans* and *P. denhartogi* that cover not only rocky surface, but also the clusters of mussels and different barnacles species. Both sponge species are encrusting a the surface of more than 40 cm^2^. *D. incrustans* is generally bright blue in color, like *D. fragilis* in the entrance of the caves in Crimea (Ereskovsky, Kovtun & Pronin, 2016). However, at the entrance of Tulenova cave, *D*. *incrustans* specimens are small and reduced in number, because this zone is completely exposed to the waves and sunlight. In the same zone of Budova cave, numerous small individuals of *Clathria cleistochela* develop.

In the middle part of the caves, a relatively hydrological calm zone with drastic reduction of incoming light, other sponge species with more thickened and soft body contribute to the diversity, like *Haliclona* spp*.* 1–3*,* and *Halichondria bowerbanki.* For *Haliclona* spp. small outstretched oscular chimneys, and long, fine outgrowths are found at the surface. The latter is similar to hydrological calm zones of Crimea underwater caves (Ereskovsky, Kovtun & Pronin, 2016). In this zone *D. incrustans* and *P. denhartogi* become small and very thin and their abundance decreases towards the inner part of caves.

So far, 87 species belonging to the classes Demospongiae and Calcarea (Table 3) are described from the Black Sea. This number is low number compared to the Mediterranean Sea, which harbors more than 650 species from all four poriferan classes (Pansini & Longo, 2003). For the coastal zone of Bulgaria some 37 species have been reported, which constitute 42.5% of the known Black Sea sponge fauna. Of these, only eight sponge species (21.6%) inhabit the shallow-water semi-submerged caves (Table 4). The use of combine genetic and morphology analyses allowed us for the first time for the Black Sea to identify two species: *Protosuberites denhartogi* and *Halichondria bowerbanki.*

**Table 4:** Sponges from marine caves from the North-East of Bulgaria and their spatial distribution in relation to the entrance to the cave.

No	Sponges	Budova	Tulenova	Temnata dupka
1	*Dysidea incrustans* (Schmidt, 1862)	0–37 m	0–33 m	0–6 m
2	*Protosuberites denhartogi* van Soest, de Kluijver, 2003	0–42 m	0–33 m	0–70 m
3	*Clathria (Microciona) cleistochela* (Topsent, 1925)	0–22 m	22–25 m	9–22 m
4	*Pione* cf *vastifica* (Hancock, 1849)	6–35 m	22–33 m	22–70 m
5	*Halichondria bowerbanki* Burton, 1930	20–30 m	22–33 m	22–28 m
6	*Haliclona* sp*.* 1	20–28 m	25–33 m	22–28 m
7	*Haliclona* sp*.* 2	31–42 m	22–33 m	22–70 m
8	*Haliclona* sp. 3	No	25 m	12–15 m

In general, semi-submerged caves host a characteristic lower abundance of sponge diversity in comparison to fully submerged marine caves. This is rather a consequence of higher hydrodynamics and instable environmental conditions than oligotrophy, which is rare in this type of caves. Sponge species composition in caves of Bulgaria as well as Crimea is different from those studied in the Mediterranean (Pouliquen, 1972; Balduzzi et al., 1989; Harmelin & Vacelet, 1997; Arko-Pijevac et al., 2001; Gerovasileiou & Voultsiadou, 2012; Gerovasileiou & Voultsiadou, 2016; Manconi et al., 2013), probably due to the geographic isolation of the Black Sea and the differences in the hydro-chemical parameters of the milieu.

The study of sponges from caves of the Black Sea is just at its beginning. At present, only two areas have been surveyed: the West of Crimea and the North-East of Bulgaria. In total 15 species (17.2% from all Black Sea sponges) were reported from caves, of which only *Pione* cf *vastifica* and *Clathria cheilochela* inhabit the caves of both regions. This percentage is rather small compared to the 311 species (45.7% of the Mediterranean poriferans), representing all four sponge classes, which have been recorded in Mediterranean marine caves (Gerovasileiou & Voultsiadou, 2012).

The present study of underwater cave sponge assemblages in the Black Sea fills regional knowledge gaps for a habitat of special conservation interest. The results of our study highlight the need for (1) further study of the Black Sea underwater caves, and (2) a deep revision of the sponge fauna present in this sea.

##  Supplemental Information

10.7717/peerj.4596/supp-1 File S1 Budova Cave

10.7717/peerj.4596/supp-2 File S2 Tulenova Cave

10.7717/peerj.4596/supp-3 File S3 Temnata dupka Cave

## Additional Information and Declarations

### Data Availability

The following information was supplied regarding data availability: Sequences are deposited in NCBI Genbank under accession numbers MH157894–MH157912, and with location data, specimen photos (including *in situ* photos) and additional morphological information in the form of thin section photos and spicule preparations in the Sponge Barcoding Database (SBD, http://www.spongebarcoding.org, Wörheide & Erpenbeck, 2007), accession numbers SBD #1758–1776.
